# Supplementary material for: Learning curve and surgical outcome of robotic assisted colorectal surgery with ERAS program
Source: Sci Rep. 2022 Nov 29;12:20566. doi: 10.1038/s41598-022-24665-w (PMC9709162; doi:10.1038/s41598-022-24665-w)
Supplement: Supplementary file 2 — Supplementary Information 2. [file 41598_2022_24665_MOESM2_ESM.zip › RAL Raw data-Ñ[▒K/126/OP_ñw╝╨░O▒Kñσ.pdf]

正式摘要(電子簽章：000000230014247)

W52 / W52

臺中榮民總醫院

Operation Note

[redacted]

- 手術日期：2021/11/18
- 手術醫師：VS 蔣鋒帆，CR 陳瑋夫，R 蔡濬樟
- Anesthesia : GE
- Position : Lithotomy
- Aseptic agents : Soap

Diagnosis

- Pre-operative diagnosis : Malignant tumor of rectum
- Post-operative diagnosis : Ditto.s/p Robotic TME+loop ileostomy

Surgical Indication

- for curative resection

Operation

- 1. Robotic (XI) assisted TME+ loop ileostomy
  2. Intraoperative colonoscopy : (-).
  3. High ligation (+) : At IMA root (Via robotic clips). IMV was ligated via endoclip.
  4. Take down from the splenic flexure : (-)
  5. Proximal cut end: > 10 cm, distal cut end: 1.5 cm
  6. No drainage tube was inserted
  7. The residual proximal D colon was sutured to distal rectal stump via hand sewn with 1-0 Vicryl
  8. Loop ileostomy (About 15~20cm from IC valve) was done over RLQ of abdomen

Finding

- 1. A 2x2 cm ulcerative tumor at dentate line, FAV about 2cm
  2. No other gross tumor nor polyps was found
  3. There was no obvious visible liver metastasis, no visible peritoneal seeding
  4. No obvious ascites was noted

Procedure

- 1. General endotracheal anesthesia was induced and then the patient was placed in modified Trendelenburg lithotomy position.
  2. Preoperative antibiotics were given. A Foley catheter and nasogastric tube were placed.
  3. The abdomen was prepped and draped in standard sterile fashion.
  4. A small vertical incision was made above the umbilicus and a 8 mm trocar was inserted into the peritoneal cavity by open method. Peumoperitoneum was established with CO2 inflation.
  5. Laparoscopy was inserted and then other two 8mm trocars were inserted over LUQ, RLQ of abdomen. Another 12mm trocars was inserted over RLQ area, and the other assist-port 12mm troca was inserted on RUQ. The operative findings were mentioned as above.
  6. The sigmoid colon was held by grasper and the peritoneum overlying the mesentery was scored with electrocautery.
  7. The inferior mesentery artery was identified, ligated above the left colic artery with robotic clips, and transected. IMV was ligated via endoclip and then also transected.
  8. Dissection was done above the Gerota's fascia and toward the peritoneal attachments.
  9. The colon was mobilized from retroperitoneum overlying the sacral promontory and into the pelvis.

  10. Distal descending colon and s-colon were freely mobilized after the mesoclon was all dissected.

- 11. The incision was then extended laterally and medially across the peritoneal reflection.
  12. The mesorectum was then mobilized using sharp dissection over the level of pelvic floor muscle. Then rectum was mobilized as possible.
  13. DRE was performed at the same time to make sure the desired resection line is adequate or not but due to super low position of the tumor, the residual proximal D colon was pulled through per anus and sutured to distal rectal stump via hand sewn with 1-0 Vicryl
  14. Then we came back to laparoscope and the desired ileum site (About 15~20cm from IC valve) was selected for further ostomy construction.
  15. The bleeder was checked thorughly again.
  16. The 12mm trochar wound over the RLQ area was further extended to creat the site for loop ileostomy.
  17. The ileum was retracted from the desired ostomy site. A window was created under the ileum by dividing the mesentery adjacent to the bowel wall with electrical cautery carefully, with a plastic bridge in placed.
  18. The trochar fascia wounds were all then closed with interrupted sutures via 1-O Vicryl.
  19. Massive N/S was applied to irrigate the wound.
  20. The wound was further closed with 3-O Vicryl, and the skin was applied with SurgiSeal.
  21. Moist pads were placed and the ileum was opened. Succus and air were aspirated.
  22. The ileostomy was matured with multiple interrupted mucocutaneous suture via 4-0 Monocryl.
  23. The ileostomy was well formed and viable. An ostomy bag was placed
  24. The patient tolerated the procedure well and was brought to the POR in stable condition.
- Estimated blood loss: minimum
- 器械數量: 正確
- 紗布數量: 正確

Image

- PAINT
  - abdomen

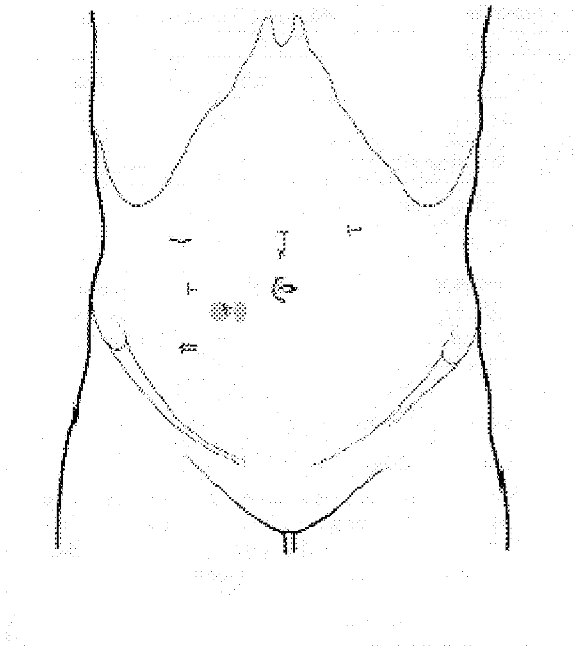

  - colon & rectum

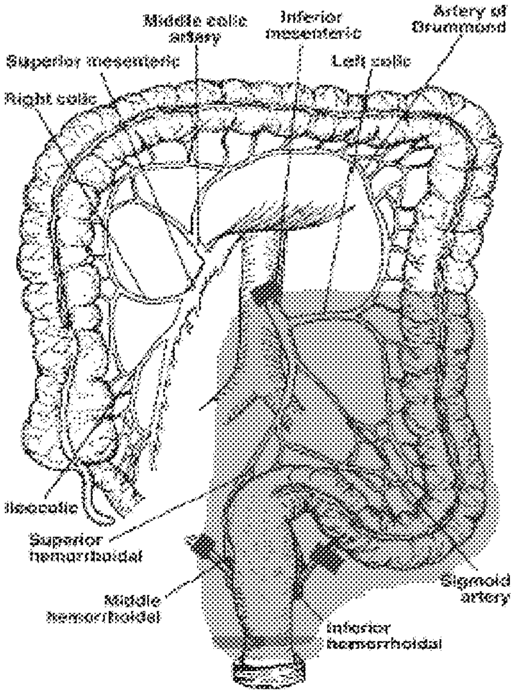

主任 2062F蔣鋒帆 / 醫師 2310K陳璿夫

正式摘要(電子簽章：**000000230014247)**

W52 / W52

doctorRank:B
